# Student nurse anesthetists’ learning during clinical practice: a mixed-methods study

**DOI:** 10.1186/s12909-026-09944-8

**Published:** 2026-07-17

**Authors:** Jakob Hedlund, Karin Blomberg, Hans Hjelmqvist, Maria Jaensson

**Affiliations:** 1 Department of Anesthesia and Intensive Care, Örebro University Hospital, Region Örebro County, Örebro, Sweden; 2 Faculty of Medicine and Health, School of Health Sciences, Örebro University, Örebro, Sweden; 3 Faculty of Medicine and Health, School of Medical Sciences, Örebro University, Örebro, Sweden

**Keywords:** Clinical practice, Learning, Learning environment, Operating room, Student Nurse Anesthetist, Mixed-Methods

## Abstract

**Background:**

Clinical practice is essential for student nurse anesthetists to integrate theory with practical skills; however, the demanding clinical environment can impact both stress and learning outcomes. Responsibilities and expectations differ across healthcare systems, and research in the Swedish context remains limited. Addressing this gap is essential for understanding how clinical practice contributes to the development of professional readiness and competence. This study, therefore, aimed to describe the learning experiences of students and newly graduated nurse anesthetists, with a focus on the clinical learning environment and the stress it entails.

**Methods:**

A convergent parallel mixed-methods design was used. In the quantitative strand, student nurse anesthetists were recruited using convenience sampling to complete a web-based survey that included the two instruments, “Clinical Learning Environment Supervision and Nurse Teacher evaluation scale” and “Perceived Stress Scale” (with 10 items). Data were analyzed with descriptive statistics, while free-text answers were analyzed using summative content analysis and word frequencies. In the qualitative strand, newly graduated nurse anesthetists were recruited using purposive sampling to participate in individual semi-structured interviews. Data were analyzed with content analysis. After separate analysis, the pillar integration process was used to integrate quantitative and qualitative data.

**Results:**

The analysis included data from 63 surveys (63/225, 28%) and 13 interviews. The integration of data from both strands yielded two divergent themes, “An unfamiliar but supportive learning environment” and “Experience of stress,” and two convergent themes, “Supervisors, a facilitator to learning” and “Dynamic learning situations.”

**Conclusion:**

Despite the challenges of adapting to new knowledge contexts, participants reported high satisfaction with the clinical learning environment, as shown by elevated scores in the Clinical Learning Environment Supervision and Nurse Teacher evaluation scale. Clinical supervisors played a pivotal role in supporting learning, and the environment’s pedagogical flexibility enabled both individual and collaborative approaches. Although stress levels varied, the average Perceived Stress Scale score remained low.

**Supplementary Information:**

The online version contains supplementary material available at 10.1186/s12909-026-09944-8.

## Background

In previous research, student nurse anesthetists (SNAs) viewed clinical practice in the operating room (OR) as the most important opportunity to develop skills for their future practice [1]. However, the OR as a learning environment can be complex and involves much technology [2], interprofessional teamwork [3], and demands for time and cost efficiency [4]. Requirements for students’ clinical readiness have previously been described as a willingness to learn, including taking responsibility for their own learning, asking questions, and receiving constructive feedback [5, 6]. Additionally, acting professionally and using effective, respectful communication are essential components for students to be prepared for clinical practice [5, 6]. In a Swedish context, students’ clinical preparedness can also be understood in relation to the national learning outcomes described in the Higher Education Ordinance [7], including requirements for clinical competence, patient safety, and professional judgment [7, 8]. For nurse anesthetist (NA) education, these competencies are further specified in program syllabi and professional competence descriptions [9].

Students’ experiences in the clinical learning environment can influence their learning outcomes [10, 11]. Although clinical practice is grounded in theory, the transition from classroom learning to clinical settings can create a gap between theory and practice [12]. Previous research has shown that SNAs describe clinical practice as a vulnerable situation [1], characterized by higher demands than expected and information overload [6]. Furthermore, SNAs have reported that their well-being is usually overlooked during clinical practice [13].

Being admitted to a nurse anesthesia program (NAP) and taking on the role of SNA is a stressful situation [13–17]. Stress can motivate learning, but it can also have adverse effects if it becomes overwhelming [18]. Early studies, dating back to the 1980s and 1990s, reported stress among SNAs and recognized that admission to a NAP involves social situations that can contribute to stress, such as loss of income and social activities [16, 17]. Additionally, clinical stressors, such as role ambiguity, first-time events, and the initial fear of clinical errors, as well as increased responsibility later, have been reported [16, 17]. The early finding aligns with more recent research from the 2010s, which is by now also relatively dated [14, 19]. Nevertheless, a study conducted in the 2020s on the same subject reported a similar pattern, although the contextual conditions differed from those of the present study [13]. Furthermore, the responsibilities in the NA profession, which students are trained for, vary internationally [20, 21], as does the length of the NAP [20, 22]. Worldwide, NAs operate with different levels of independence. In a European context, this can mean working either under direct supervision, where an anesthesiologist is present in the OR, or under indirect supervision, where the anesthesiologist is not physically present but available if needed [20]. In contrast, NAs in some US states work independently without supervision from an anesthesiologist [23]. Differences among countries are also evident in formal training. For example, in Europe, SNAs receive either no formal training or up to four years of training with varying amounts of clinical practice, depending on the country [20, 22]. Outside Europe, in the USA, SNAs must complete at least 2,000 h of clinical practice and 650 cases [24]. These differences suggest that learning is influenced by the specific context. In Sweden, SNAs complete a one-year master’s program (60 credits), with clinical practice lasting from 9 to 15 weeks due to the lack of a national standard [7]. While clinical practice is crucial for developing skills, there is limited research on SNAs’ learning experiences within the Swedish context. Addressing this gap is essential for understanding how clinical practice contributes to the development of professional readiness and competence. Therefore, this study aimed to describe the learning situation during clinical practice, focusing on the learning environment and stress from the perspective of SNAs and newly graduated NAs. The following research questions were included:

- How do SNAs evaluate their clinical learning environment and perceive stress during clinical practice?
- How do newly graduated NAs describe their learning experiences during clinical practice as former students?
- How do the perspectives of SNAs and NAs align and differ?

## Methods

### Design

**Fig. 1:**
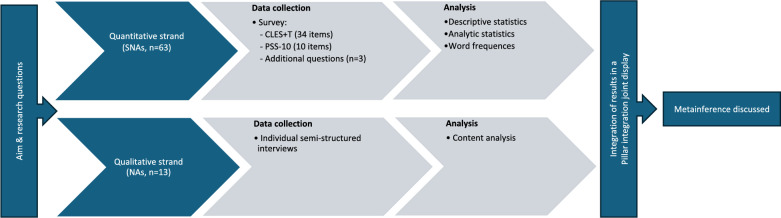
Flowchart of the methodological process

This study adopts a mixed-methods approach with a convergent parallel design (Fig. 1) [25] and combines the results using the pillar integration process (PIP) [26]. Relying solely on either quantitative or qualitative methods was deemed insufficient to fully understand the complexity of student learning in the OR. Integrating both quantitative and qualitative data provided a more comprehensive understanding of SNAs’ clinical learning experiences in the OR.

### Quantitative strand (SNAs)

#### Setting and participants

In the quantitative strand, participants were recruited nationally through convenience sampling. SNAs enrolled in a Swedish NAP were invited to complete a web-based survey about their clinical practice in an OR setting. At the time of data collection, 13 universities offered NAPs, with clinical placements occurring throughout the year. The survey was sent to 12 of these universities after one university declined to participate in the study.

#### Data collection

Quantitative data were collected through a national web-based survey using two instruments. Invitations were sent to SNAs via program directors, followed by two reminders. The invitation included written information and a survey link, with consent indicated by selecting a box before participation. The survey was accessible on both computers and mobile devices.

Oral information about the study was provided to potential participants by the first author via video conferencing platforms. When possible, this was done in connection with SNAs’ classroom teaching before the start of clinical practice. In one case, a recorded video with oral information was uploaded by the program director to the SNAs’ digital learning platform.

The instruments used were the Swedish version of the Clinical Learning Environment Supervision and Nurse Teacher (CLES + T) evaluation scale and the Perceived Stress Scale with 10 items (PSS-10). These were converted to an electronic format using ORU-Survey (Artologic Survey&Report). Due to varying clinical practice periods, the survey was sent at the end of each university’s final placement period. The first invitation was sent two weeks in advance, followed by reminders during the final week and the week after. The survey was conducted between November 2020 and June 2021.

The CLES + T scale was initially developed by Saarikoski et al. in 2008 [27] and has been translated into other languages, including Swedish [28]. It consists of 34 items and covers five dimensions: pedagogical atmosphere (*n* = 9 items), leadership style of the ward manager (*n* = 4 items), premises of nursing on the ward (*n* = 4 items), supervisory relationship (*n* = 8 items), and role of the nurse teacher (*n* = 9 items). Each item was answered with a 5-point Likert-scale: (1) completely disagree, (2) disagree to some extent, (3) neither agree nor disagree, (4) agree to some extent, and (5) fully agree. The Swedish version of the CLES + T scale has demonstrated satisfactory psychometric properties [28, 29].

PSS-10 is an instrument originally developed by Cohen et al. (1983) [30] that measures the extent to which life situations are perceived as stressful. The instrument consists of 10 items that begin with “In the last month, how often have you… .” Each item was answered on a 5-point Likert-scale: (0) never, (1) almost never, (2) sometimes, (3) fairly often, and (4) very often. The Swedish version of PSS-10 has demonstrated satisfactory psychometric properties [31].

The authors added three questions to the survey: “To what extent has your clinical practice affected your experience of stress?” (answered on a 4-point Likert scale: (1) not at all, (2) a little, (3) moderately, or (4) to a great extent); “How satisfied are you with your clinical practice?” (answered on a 4-point Likert scale: (1) dissatisfied, (2) partially dissatisfied, (3) satisfied, or (4) very satisfied); and “What has been important to your learning process during clinical practice?” (an open question). Participants also had the option to provide additional comments in free text.

#### Analysis

The analyses were conducted using descriptive and analytic statistics. For the quantitative data collection, we calculated the sample size based on an assumption of a sample-to-item ratio of 5:1, which included five responses per item [32]. With 47 items included in the survey, we assumed that 235 respondents would be sufficient. However, it was estimated that we would likely not achieve 235 respondents, as only about 240 SNAs are admitted in Sweden each year, assuming that every position is filled. With an estimated response rate of 65%, this would yield a maximum of 153 participants.

Demographic data were presented as the mean ± standard deviation (SD) for continuous variables and as the frequency for categorical variables (Table 2). The CLES + T scale was presented with the mean and SD for each subdimension to enable a comparison with other studies using this instrument [33–35]. Additionally, internal consistency, as measured by Cronbach’s alpha, was analyzed for the CLES + T scale. The PSS-10 was presented with the mean. In the PSS-10 instrument, six of the 10 items were negatively worded, while four were positively worded. Based on the user’s manual, the positive items in the PSS-10 (i.e., items four, five, seven, and eight) were reversed before analysis [30].

As mentioned above, two of the additional questions were answered on an ordinal scale. Of these, the question, “To what extent has your clinical practice affected your experience of stress?” was dichotomized into two groups (not at all/low degree and moderately/to high degree).

To compare groups, the Mann-Whitney U test was used to investigate differences in terms of sex, age, and whether their education was externally financed. One question (the OR’s size) was excluded from the analysis due to weakness in reported data. The demographic variable of age was dichotomized into two groups based on the median, with a cutoff at 32 years.

Statistical analyses were conducted using IBM SPSS Statistics (v. 28). The significance level was set to *p* < 0.05.

Free-text answers in the survey were analyzed using a summative content analysis [36]. Words and sentences related to learning during clinical practice were identified and highlighted. Then, the word count among the identified words was computed to identify patterns.

### Qualitative strand (NAs)

#### Setting and participants

In the qualitative strand, participants were recruited from anesthesia departments in six out of 21 Swedish counties to ensure a variety of hospital sizes, including both urban and rural areas. A purposive sample was selected for semi-structured interviews, focusing on NAs with no more than 2 years of experience.

#### Data collection

Qualitative data were collected through individual semi-structured interviews. A webpage was created for this study, where NAs could register their interest in participation. This weblink was distributed in anesthesia departments via the manager to NAs who met the criteria. When visiting the webpage, potential participants received brief information about the study and were able to leave their contact information. They were then contacted by the first author to receive verbal information; they were also mailed printed materials and a consent form. Before conducting the interviews, an interview guide (Appendix A) was created, including areas inspired by a previous review on SNA learning in the OR [11]. The interview guide covered topics such as SNAs’ overall experience in clinical practice, supporting and hindering factors, the learning environment, their emotional state, and ways to improve student learning. One pilot interview was conducted without any revisions to the interview guide and was therefore included in the analysis. The interview began with the prompt: “If you think back to when you were a student in the OR receiving supervision, can you tell me about your experience from your clinical practice?” The interviews (*n* = 13) took place between March 2022 and November 2022, all conducted by JH. Participants were allowed to choose the location and format of the interview, either a telephone interview (*n* = 9) or a face-to-face interview (*n* = 4). All interviews were audio recorded (Philips, Vienna, Austria, VoiceTracer DVT 8110) and transcribed by a professional transcriber. The interview length varied between 25 and 84 min (mean, 59 min).

#### Analysis

The interviews were analyzed using inductive content analysis [37]. The analysis was performed in Nvivo (v. 14). First, all interviews were read and reread multiple times to become familiar with the data. Then, units of meaning related to the aim were openly coded. Sentences that addressed the aim were labeled with descriptive tags closely matching the text. All codes were compiled into a list, and those describing similar content were grouped into subcategories. After discussions among all authors, four categories and one main category were developed by abstracting the subcategories (Table 1).

**Table 1:** Examples of the analysis process

Text	Open coding	Sub-category	Category	Main category
It was very scary in the beginning, of course, since it was a brand new environment you stepped into, after all, because it is very high-tech, there are so many rules that you’re not updated on before you start the continuing education, and how you should behave in an operation room, and what you can and what you can’t do. Both regarding sheer hygiene matters, when you should have surgical masks or not, and other things, where in the room you can be, and so on.	New rules	Context	To acclimatize to a new environment	To find your truth
… as a first internship, it turned out that we were only two students per one supervisor, so it was more like… we got every second patient…	Every other patient	Peer learning	To balance individual and collaborative learning	

### Data integration

To answer the question of how the results from the two strands converge and diverge, the quantitative and qualitative results were integrated and displayed together (Table 4) using the four stages of PIP: listing, matching, checking, and pillar building [26]. The integration process started with listing the qualitative categories from the content analysis in a qualitative (qual) category column, with supporting raw data placed in a qual code column, relevant to understanding SNAs’ clinical learning situation. The results from the quantitative (quant) strand were then matched with the listed qualitative data in a quant data and quant categories column, containing content that reflected the initial listed data. The joint display was compared horizontally to determine whether the rows reflected each other. After verifying the matching of qualitative and quantitative data, the findings were compared and contrasted to identify common and differing themes. The development of divergent themes indicated a discrepancy, whereas convergent themes indicated alignment between qualitative and quantitative data. The initial pillar-building process was performed by the first author, and the process was discussed among all authors until consensus was reached.

### Ethical considerations

The study received approval from the Swedish Ethical Review Authority (Dnr 2020–00686, 2021-06027-01, 2022-02095-02). Permission to use the CLES + T and the PSS-10 was obtained from the respective copyright holders. Participation was voluntary, and the survey was completed anonymously. Interviews took place at locations chosen by the participants, conducted by interviewers who had no prior relationship with them. Data were stored securely and accessible only to the authors.

## Results

Quantitative and qualitative data were collected from two distinct samples (Table 2) and are shown separately below. This section begins with the quantitative strand (SNAs), followed by the qualitative strand (newly graduated NAs).

**Table 2:** Characteristics of participants (*n* = 76)

	Survey, SNAs^b^ (*n* = 63)	Interviews, NAs^c^ (*n* = 13)
Sex		
Men, *n* (%)	21 (33.3)	8 (61.5)
Women, *n* (%)	42 (66.7)	5 (38.5)
Age, yrs		
Mean	33	35.5
Median	32	36
Min–max	25–48	26–49
Working experience as RN^a^, yrs		
Mean	6.7	6.6
Median	6	5
Min–max	1–21	1–14
Weeks with clinical practice, median (min–max)	13 (8–17)	13 (11–15)
Rate of study		
Full-time studies, *n* (%)	63 (100)	
Part-time studies, *n* (%)	0 (0)	
Education paid by the employer		
Yes, *n* (%)	44 (69.8)	
No, *n* (%)	19 (30.2)	

^a^RN, registered nurse; ^b^SNA, student nurse anesthetist; ^c^NA, nurse anesthetist

### Quantitative strand (SNAs)

The overall response rate for the survey was 28% (*n* = 63/225). There were no unit non-responses. In the CLES + T, some item non-responses were missing at random; five items had two missing responses, and eight items had one missing response. In the PSS-10, there were no item non-responses. No statistically significant differences were found in the CLES + T subdimensions when comparing groups such as sex, age, and whether their education was externally financed.

#### CLES + T

Overall, the participants reported a mean CLES + T score of 4.15 (SD 0.53). Additionally, high mean scores were reported for the items “The ward can be regarded as a good learning environment” and “There were sufficient meaningful learning situations,” which were 4.56 (SD 0.74) and 4.46 (SD 0.86), respectively.

The subdimension with the highest reported mean score was “Supervisory relationship,” at 4.49 (SD 0.81). The individual items included in this subdimension were: “Overall, I am satisfied with the supervision I received” (4.40 [SD 1.03]), “I felt that I received individual supervision” (4.60 [SD 0.77]), and “I continuously received feedback from my supervisor” (4.33 [SD 0.98]).

The lowest mean score was for the subdimension “Leadership style of the ward,” at 3.59 (SD 0.83). For the sub-dimensions, Cronbach’s alpha ranged from 0.77 to 0.96 (Table 3).

**Table 3:** Score for the CLES + T sub-dimensions

Sub-dimensions	Mean	SD^a^	Cronbach’s alpha
Pedagogical atmosphere 9 items	4.31	0.59	0.89
Leadership style of the ward manager 4 items	3.59	0.83	0.79
Premises of nursing on the ward 4 items	3.90	0.75	0.77
Supervisory relationship 8 items	4.49	0.81	0.96
Role of the nurse teacher 9 items	4.04	0.76	0.88
Total score for CLES + T	4.15	0.53	0.92

^a^*SD* standard deviation

#### PSS-10

The mean sum score for the PSS-10 was 13.87 (SD 6.47). Women reported a significantly higher mean score (SD) than men, at 15.24 (SD 6.32) versus 11.14 (SD 6.04) (*p* = 0.025). No statistical difference in perceived stress was found based on age or externally financed education.

#### Additional questions

Regarding the additional question, “How satisfied are you with your clinical practice?” 89% (56/63) stated that they were satisfied with their clinical practice. For the question, “To what extent has your clinical practice affected your experience of stress?” 51% (32/63) said that their clinical practice had contributed to their experience of stress.

#### Free-text answers

In the summative analysis of the free-text responses to the question, “What has been important to your learning process during clinical practice?” the word “supervisor” appeared most frequently, at 22% (38/173 codes), followed by “team,” at 11% (19/173 codes), and “independence,” at 10% (17/173 codes).

### Qualitative strand (NAs)

The qualitative result is shown with one main category, “To find your truth,” and four subcategories: “To acclimate to a new environment,” “To be tutored,” “To thrive under pressure,” and “To balance individual and collaborative learning.”

#### To find your truth

The main category, “To find your truth,” describes satisfaction with clinical practice and the OR as a learning environment. However, carrying out clinical practice requires facing a variety of different tensions. This includes the interaction between old knowledge and new experiences, the importance of guidance from supervisors versus the desire for independence, parallel clinical and didactic learning processes, and balancing individual and collaborative learning.

#### To acclimatize to a new environment

The participants entered the NAP with previous experience as registered nurses. Starting clinical practice was seen as a new, energy-consuming situation filled with many new impressions. Sometimes, it was difficult to understand what was happening and how everything was connected. In their former student role, shifting from a familiar workplace with established routines to a new environment was stressful. Participants said they were not prepared for such a controlled, highly technological setting with specific rules, many different drugs, and advanced terminology. Additionally, the routines in surgical departments within the operating ward could vary. Participants described experiencing a wide range of feelings during clinical practice, from fun and excitement to feeling awful and crying daily.

The first period with clinical practice is about survival, to get there and make the day. (Participant 12)

Time was a recurring topic in the interviews, and participants described time pressure as a barrier to learning. There is an estimated time for each step of a surgery, and the participants had to keep up with their supervisor’s pace. Learning opportunities were sometimes said to be lost because there wasn’t enough time, and there was a need to keep everything “rolling.”

… there were people [staff] who said straight out, “Maybe you shouldn’t have students in that particular room, because here it really has to go fast,” and who complained a lot over things that went slowly. (Participant 6)

#### To be tutored

Supervisors played a vital role in learning, and the student–supervisor relationship was seen as necessary to support learning. In the interviews, participants shared how different supervisors performed anesthesia in varied ways. High supervisor continuity was seen as a helpful factor for learning, but it could also be a problem if students were asked the same questions repeatedly by the supervisor. Conversely, low supervisor continuity was viewed as a barrier to learning; with a new supervisor, students had to start from scratch, show their current knowledge, and earn the supervisor’s trust.

Participants wanted supervisors to view them as future colleagues. The desirable traits of a supervisor mentioned in the interviews included being familiar with the student’s current knowledge level and adjusting the supervision accordingly. It was also preferred for supervisors to be patient, stress-tolerant, calm, confident in their professional role, interested in teaching, encouraging, able to see students’ potential, capable of explaining “why,” up to date with evidence-based practices, and able to guide students verbally without immediately intervening or taking over. It was seen as valuable to have a space to make mistakes within acceptable limits, while maintaining patient safety and avoiding unnecessary risks.

She [the supervisor] was great the first time, but then, this last stage, when she was really supposed to let me do everything by myself, then I thought it was like, it was almost daily, “Can you step aside [name], because I really need to, I want to do this myself now.” (Participant 9).

Participants also reported receiving individual verbal feedback from their supervisor. This was sometimes given directly during case management and sometimes at the end of the day. Verbal feedback could be initiated by either the supervisor or the participants. Written feedback was mainly provided during assessments. However, one participant mentioned using a logbook started by the institution, where students wrote reflections from the day and supervisors added notes or comments.

#### To thrive under pressure

In the interviews, participants described being enthusiastic about clinical practice but also needing to manage simultaneous learning processes. Before clinical practice, they outlined preparations as involving theoretical courses and hands-on experience in clinical training centers. They felt excited and eager to turn theory into practice and “get the knowledge out in their hands.” Participants mentioned feeling like they always needed to be prepared and alert because they never knew what questions they might face. However, despite expressing the need for theoretical knowledge, they found it challenging to balance theoretical courses with clinical practice. Occasionally, the workload was described as more than full-time, making it difficult for participants to fully focus on their clinical learning.

The interviews revealed a perception of high self-imposed requirements that were lower in the first semester and increased in the second semester, when the participants were expected to manage cases more independently. Additionally, learning was not limited to the OR; it continued through discussions with fellow students in the break room and during lunches. Furthermore, the participants had to study at home after finishing their day in clinical practice.

#### To balance individual and collaborative learning

Participants described experiencing a balance and tension between individual learning and collaborative learning (i.e., peer learning [PL]). They discussed actions through which they took responsibility for their learning, such as communicating their individual learning needs to the supervisor, asking questions, and actively seeking information. Not being involved in the placement process was seen as a hindrance, and participants explained how they asked to be placed based on their learning needs, for example, to encounter more laryngeal masks or intubations. Additional actions included preparing for the next day’s patient cases the day before and tracking their learning process using a checklist.

Besides individual supervision, participants also shared their experiences with PL during clinical practice. The amount of PL and the experiences shared varied. In some cases, participants practiced PL more frequently, sometimes once a week and other times every other week. The positive aspects of a PL approach included feeling safer, finding it easier, and experiencing less stress when asking a fellow student questions instead of the supervisor. One drawback of PL was that participants and fellow students could have different levels of knowledge. For example, participants mentioned being unable to learn from PL because of different knowledge backgrounds. As a result, in some cases, participants felt they needed to step back in favor of their peers. Other negative aspects of PL included a feeling of being compared with peers. In some cases, participants stated that it would be better to have PL at the beginning of clinical practice. One participant who had PL at the end of clinical practice described it as stressful because they wanted extensive training before graduation in order to be better prepared to start working.

### Merging

The integration of the two data strands using PIP [26] resulted in four themes (Table 4), including two divergent themes, “An unfamiliar but supportive learning environment” and “Experience of stress,” and two convergent themes, “Supervisors, a facilitator to learning,” and “Dynamic learning situations.”

**Table 4:** Pillar integration joint display

QUAL codes	QUAL categories	Pillar building theme	QUANT categories	QUANT data
	
“It’s a different world compared to the rest of healthcare” “It’s a new situation even if you’ve been working for a while” “You are under time pressure. You have a set time for how long the anesthesia should take” “I had to keep up with the tempo of the supervisors”	To acclimatize to a new environment	Divergent theme: An unfamiliar but supportive learning environment	Participants rate the OR as a good learning environment.	89% were satisfied with their clinical practice. Total mean score for CLES + T was 4.15 (SD 0.53). Total mean for item 9^a^ was 4.56 (SD 0.74). Total mean for item 7^b^ was 4.46 (SD 0.86).
“Supervisors possess a key role in the outcome of learning during clinical practice” “Supervisors should be confident in their professional role and have a willingness to supervise” “You learn more when you have to think and act on your own” ”It’s easier to learn from your mistakes, and if you are assisted to reflect afterwards”	To be tutored	Convergent theme: Supervisors, a facilitator to learning	Supervisors are a facilitator to student learning.	The word “supervisor” appeared most frequently as the most important facilitator to clinical learning. Total mean for item 21^c^ was 4.40 (SD 1.03). Total mean for item 19^d^ was 4.60 (SD 0.77).
“You had school assignments concurrently with clinical placement” “It felt like 150% workload was expected during the clinical practice”	To thrive under pressure	Divergent theme: Experience of stress	Although half of participants state clinical practice contributes to their experience of stress, they score low levels of stress in PSS-10.	51% stated clinical practice had contributed to their experience of stress. Total mean sum score for PSS10 was 13.87(SD 6.47). Women reported a significantly higher mean score than men: 15.24 (SD 6.32) vs. 11.14 (SD 6.04) (*p* = 0.025).
“I tried to take responsibility for my learning, and I pointed out, *I want to know…*” “We were two students on one supervisor, so we got every other patient during first period with clinical practice” “I did the last period in peer learning”	To balance individual and collaborative learning	Convergent theme: Dynamic learning situations	There was variation in the execution of learning situations.	Total mean for item 8 ^e^ was 4.40 (SD 0.69).

^a^The ward can be regarded as a good learning environment; ^b^There were sufficient meaningful learning situation; ^c^Overall, I am satisfied with the supervision I received; ^d^I felt that I received individual supervision; ^e^The learning situations were multidimensional in terms of content

## Discussion

**Fig. 2:**
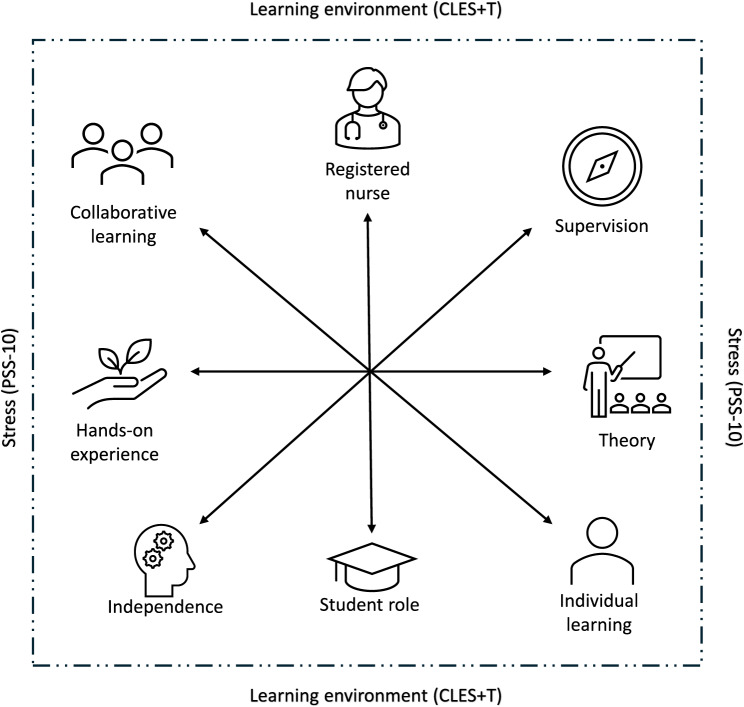
Illustration of the main findings from the pillar building themes

This study confirms previous findings on learning during clinical practice. Prior research has mainly focused on other countries, student groups, or broader nursing education contexts. Our study advances this field by focusing on the clinical training of SNAs in Sweden. It also offers insights that are directly applicable to educators, supervisors, and curriculum developers in this specialty. A description of the SNAs’ clinical learning experience is provided through four themes (Table 4), which integrate quantitative and qualitative data for a more comprehensive understanding. The main findings are illustrated in Fig. 2.

### Divergent themes

In two themes, a discrepancy between quantitative and qualitative results was identified. The theme “An unfamiliar but supportive learning environment” suggests that participants were satisfied with their clinical practice and the OR as a setting for learning. However, clinical learning also presented challenges. This theme reflects a negotiation between old and new knowledge, including entering the OR—a new and unfamiliar environment despite previous healthcare experience. Although prior working experience as a registered nurse has been identified as a prerequisite to support SNAs’ clinical learning [38], returning to a student role can be difficult [6]. The timing of data collection during the COVID-19 pandemic may have affected the results, as the pandemic led to more online education and deviations from routine care. Nonetheless, the SNAs and supervisors showed resilience and creativity in creating clinical learning opportunities for the SNAs [39].

The theme “Experience of stress” refers to participants describing an overwhelming workload during clinical practice. Half of the participants (51%) reported that clinical practice contributed to their stress levels, with females experiencing significantly higher stress levels than males. However, this stress was not reflected in PSS-10 scores. The mean PSS-10 score in this study was lower than in prior research, at 13.87 (6.47) compared to 18.27 (3.50) [13]. One possible reason for the low mean score may be contextual, as this study was conducted in a different country than previous studies, reflecting differences in educational and healthcare systems. Therefore, the discrepancy between the quantitative and qualitative findings may be due to the PSS-10 lacking sufficient specificity to adequately capture the phenomenon in this context [40]. Another possible explanation is that SNAs experiencing high levels of stress did not prioritize answering the survey, increasing the risk of non-response bias [41]. Moreover, the timing of the survey, distributed at the end of clinical practice, may not have been optimal for capturing the stress experienced during clinical practice [42]. Additionally, the results highlighted the importance of theory and preparation, and integrating theory and assignments during clinical practice sometimes felt like an overwhelming workload to the participants, and recalling content from previous theoretical courses can contribute to feelings of being overwhelmed [6].

### Convergent themes

The role of the supervisor was emphasized as important in the theme “Supervisors, a facilitator to learning.” The word “supervisor” was the most frequently used term in the summative analysis when participants were asked what had been important to their clinical learning. The essential role of supervisors was also highlighted in the interviews; additionally, in the survey, participants rated themselves as satisfied with the clinical supervision they received. However, the results reveal a tension between needing guidance from a supervisor and wanting to be independent and maintain a collegial relationship with the supervisor. In a previous study investigating stressors among SNAs, both a lack of autonomy and too much autonomy were identified as stressors [19]. One possible reason for our results is that the interviews for this study were conducted with recently graduated NAs, and the survey was completed during the final phase of clinical practice. This reflects the pedagogical complexity of supervision, in which support cannot be understood as static but rather as a flexible, relational process. Through the lens of scaffolding, effective supervision may involve carefully calibrating support in ways that both support the student and encourage increasing independence [43].

Lastly, in the theme “Dynamic learning situations,” the results showed how participants encountered different pedagogical approaches—that is, a balance between individual and collaborative learning. Recent research highlights peer learning (PL) as a valuable pedagogical approach in SNAs’ clinical practice, promoting both personal and professional development. PL fosters greater independence and a clearer understanding of anesthesia nursing in the OR, while also encouraging reflective learning through the assisting role [44].

Viewing SNAs’ clinical learning through a sociocultural lens, the self-regulated learning (SRL) theory can be applied. The SRL theory stresses that learners actively participate in their own learning process. This involves setting goals, monitoring progress, and reflecting on results [45]. However, the social context in which the student is situated influences this process by shaping tasks and providing feedback [46]. Hence, a student’s level of self-regulation will depend on the surrounding context [47]. Participants’ descriptions of SRL were illustrated mainly in the qualitative strand. Regarding the findings, participants engaged in goal-setting by communicating learning needs and requested placement in specific ORs to receive additional training for specific learning situations. During the monitoring process, participants used deliberate strategies in case management, such as requesting the supervisor to step aside to facilitate self-practice. Furthermore, reflections were exemplified, which could occur immediately, at day’s end, or during a break. The data reveal instances of self-reflection in students’ interpretations of feedback and their own performance. However, there is a lack of information on how these reflections were translated into subsequent strategies, so only parts of the self-regulation cycle were identified.

### Methodological considerations

A considered strength of the study is the method including both quantitative and qualitative data. However, this study is not without limitations. The low response rate in the quantitative strand limits generalizability. Non-response bias is a known risk associated with low response rates [48] and may have a negative effect on the external validity. The consequence of non-response bias is that certain perspectives may be underrepresented, which risks skewing the results and drawing incorrect conclusions. However, the absence of unit non-responses strengthens the data. The survey was distributed at the end of clinical practice to ensure participants had sufficient experience, though this timing may have affected participation [42].

Some items were perceived as less relevant by participants, particularly those related to managerial roles. The Swedish versions of the validated CLES + T scale (Cronbach’s alpha = 0.92) [28] and the 10-item PSS [31] were used to ensure reliability and reduce survey fatigue.

The authors created additional questions for the survey. Two of these were answered on a 4-point Likert scale, whereas the instruments used a 5-point Likert scale. During the design process, the authors discussed the risk of offering a middle option, since people tend to gravitate toward it [49]. A four-point Likert scale was used to avoid a neutral midpoint and to encourage respondents to indicate a directional position. The use of different response scale formats limits direct numerical comparison between instruments. However, we refrain from making comparisons, and because the additional questions were developed for this study, no prior data are available for comparison.

In the qualitative strand, a reflexive approach was employed, including discussions to clarify the perspectives shaping the process to enhance trustworthiness. This involved three individuals familiar with the OR setting (JH, HH, MJ) and one unfamiliar (KB). Furthermore, the reflexive approach, combined with detailed methodological descriptions, supports the confirmability of this study. Also, the detailed descriptions further support transferability and dependability [50]. However, the inclusion criterion—graduation within two years—may have introduced recall bias [51].

Data collection relied on program directors and managers, who distributed information to participants. These could have acted as gatekeepers [52] by targeting information to potential participants in an optimistic manner, thereby introducing selection bias. Future studies should consider more similar samples and longitudinal designs to capture the development of SNAs over time better.

## Conclusion

Participants reported high satisfaction with the clinical learning environment, as indicated by elevated CLES + T scores, despite the challenges of adapting to new knowledge contexts. Although stress levels varied, the average PSS-10 score remained low. Clinical supervisors played a pivotal role in supporting learning, and the environment’s pedagogical flexibility enabled both individual and collaborative approaches. Future research should explore how specific supervisory strategies and teaching methods influence stress and learning outcomes across diverse clinical settings.

## Supplementary Information

Supplementary Material 1.

## Data Availability

The datasets generated and/or analyzed during the current study are not publicly available due to the confidentiality of the current study. The ethical approval does not include this. The corresponding author can be contacted upon reasonable request.

## Abbreviations

CLES + T: Clinical Learning Environment Supervision and Nurse Teacher
NAPs: Nurse anesthesia programs
NAs: Nurse anesthetists
OR: Operating room
PL: Peer Learning
PSS-10: Perceived Stress Scale with 10 items
PIP: Pillar integration process
SRL: Self-regulated learning
SNAs: Student nurse anesthetists
