# Supplementary material for: Student nurse anesthetists’ learning during clinical practice: a mixed-methods study
Source: BMC Med Educ. 2026 Jul 17;26:1198. doi: 10.1186/s12909-026-09944-8 (PMC13393923; doi:10.1186/s12909-026-09944-8)
Supplement: Supplementary file 1 — Supplementary Material 1. [file 12909_2026_9944_MOESM1_ESM.docx]

**Appendix A.** Interview guide.

| If you think back to when you were a student in the OR receiving supervision, can you tell me about your experience from your clinical practice? |
| --- |
| Can you describe opportunities for your learning during clinical practice? |
| Can you describe what hindered your learning during clinical practice? |
| How did you experience the learning environment during your placement? |
| What knowledge and skills does a supervisor need to supervise nurse anesthesia students? |
| How did you feel your state of mind was during your placement? |
| Can you describe the relationship between you and your supervisor? |
| Can you tell about how you took responsibility for your own learning during the placement? |
| How did you experience the opportunity for feedback during your placement? |
| How did you experience the cooperation between the university and the clinic? |
| How can learning during the placement be improved? |
